# Macrophages: shapes and functions

**DOI:** 10.1007/s40828-022-00163-4

**Published:** 2022-03-10

**Authors:** Uwe Lendeckel, Simone Venz, Carmen Wolke

**Affiliations:** Institut für Medizinische Biochemie und Molekularbiologie, Universitätsmedizin Greifswald, Ferdinand-Sauerbruch-Straße, 17475 Greifswald, Germany

**Keywords:** M1 macrophage, M2 macrophage, TLR4 signaling, NF-κB signal transduction pathway, Oxidative burst, Phagocytosis, Reactive oxygen species

## Abstract

Macrophages are cells of the innate immune system and represent an important component of the first-line defense against pathogens and tumor cells. Here, their diverse functions in inflammation and tumor defense are described, and the mechanisms, tools, and activation pathways and states applied are presented. The main focus is on the role and origin of reactive oxygen species (ROS), the important signal pathways TLR/NF-κB, and the M1/​​M2 polarization of macrophages.

**Graphical abstract:**

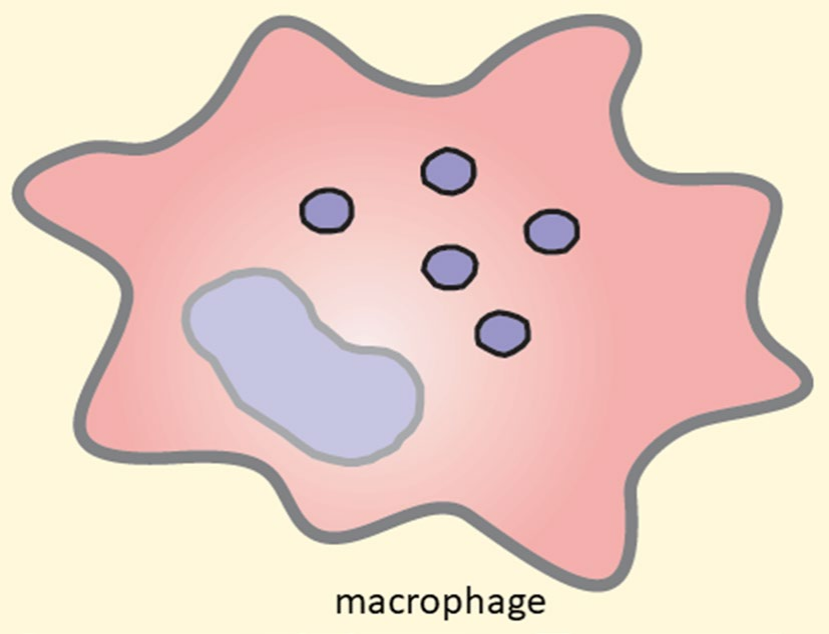

## Introduction

**Fig. 1:**
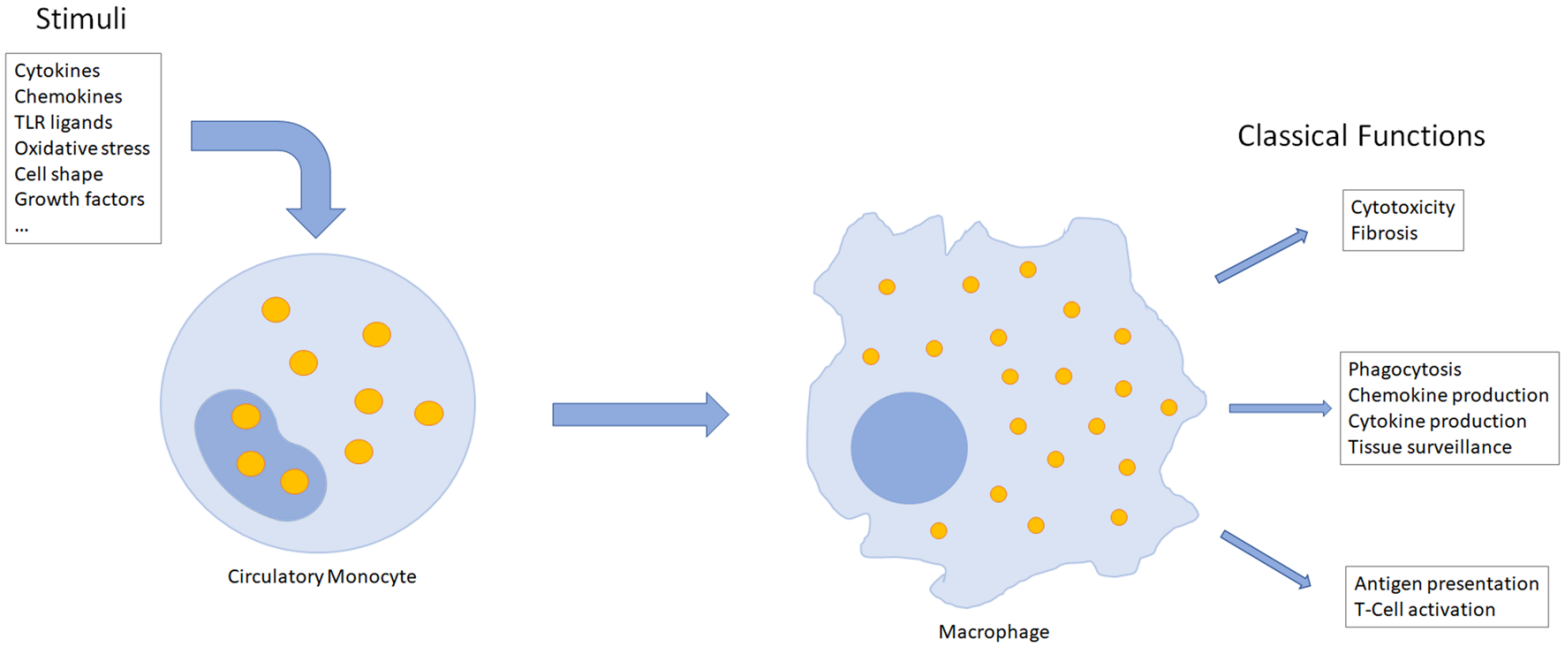
Activation of circulatory monocytes in response to variety of stimuli causes differentiation into macrophages. The shapes of macrophages are as diverse as their phenotypes and functions

**Fig. 2:**
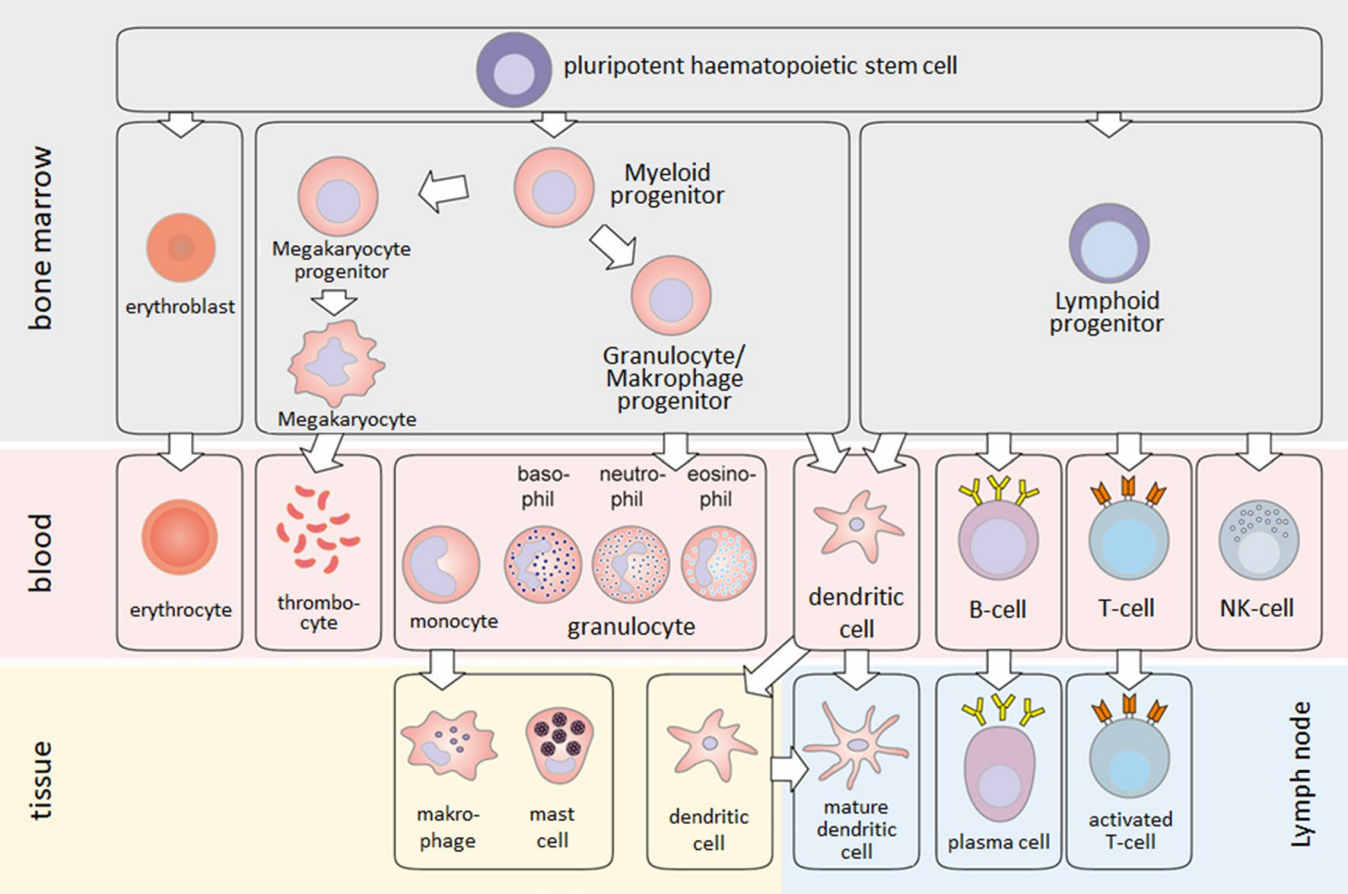
Hematopoiesis. Myeloid and lymphatic precursor cells develop from self-renewing, pluripotent hematopoietic stem cells. From these progenitor cells, lymphocytes and various myeloid cells develop, which represent the cellular arsenal of the immune system (with kind permission [75])

This lecture text has been written to introduce the nature and function of macrophages to students of biochemistry, pharmacy, chemistry, and similar subjects. Macrophages are important cells of the innate or nonspecific immune system present in all vertebrates. Like all immune cells, macrophages are derived from a pluripotent hematopoietic stem cell in the bone marrow. From this, on the one hand, lymphocytes and natural killer (NK) cells and certain dendritic cells develop via a lymphatic precursor cell. On the other hand, erythrocytes, platelets, and precursors of granulocytes and macrophages develop via a myeloid progenitor cell; monocytes, neutrophils, basophils, or eosinophils arise from the aforementioned precursors. Monocytes that migrate from the circulation into tissue mature into macrophages (Figs. 1, 2). In the circulation, the normal range of monocytes is between 2% and 8% of the white blood cell count, corresponding to 200–800 monocytes per microliter of blood. Monocytes are round cells with a kidney-shaped nucleus and are 25–30 µm in size. Upon maturation into macrophages, they assume different shapes depending on their functionality (see “M1/M2 macrophage polarization”) and usually become even larger. Macrophages can be characterized by surface markers or cellular markers. These are well established, e.g., to distinguish the different phenotypes resulting from macrophage polarization (Table 3). However, because of their common ancestry, these markers are shared to a large extent with dendritic or corresponding microglia cell subpopulations [7, 36, 63, 68].

From mid-gestation throughout life, macrophages are present in all tissues. Depending on the tissue in which they reside, macrophages posses different shapes, e.g., Kupffer cells in the liver or Langerhans cells in the skin (Table 1). In mice, macrophages present in many tissues, including skin, liver, kidney, and brain, originate from the yolk sac or the fetal liver. In adulthood, in the absence of stimulatory factors, circulating monocytes do not substantially contribute to the tissue content of macrophages. Other tissues, such as the heart and gastrointestinal tract, recruit monocytic precursors to a greater extent where immigrating monocytes differentiate into resident macrophages. In adult life, resident macrophages in many tissues represent a mix of cells originating during development and from circulating monocytic precursors. In adult humans, however, due to the extended life span, the majority of tissue macrophages seems to be recruited from the circulation [4, 24, 46]. Macrophages function in the defense against pathogens and in the clearance of old, senescent, or dead cells, but also fulfill important functions in tissue homeostasis and repair, e.g., wound healing and muscle regeneration [25]. Various macrophage populations exist that play distinct and non-redundant roles in fibrosis, tissue repair, and regeneration [26, 69]. It still remains to be established, however, whether resident or recruited macrophages represent functionally different subsets or are able to assume all possible states depending on the cellular/tissue microenvironment.

**Table 1:** Different types of tissue-resident macrophages

Tissue	Macrophage
Liver	Kupffer cells
Lung	Alveolar macrophages, pneumocytes type II
Skin	Langerhans cells
Serous cavities	Serous macrophages
Connective tissue	Histiocytes
Joints/cartilage	Synovial cells (type A)
Bone	Osteoclasts
Kidney	Mesangium macrophages
Brain	Microglia

Macrophages are an important part of the first line of defense. During infection, inflammation, or tissue injury, they can follow chemotactic signals to migrate to the damaged tissue/inflammatory site, where they ingest pathogens and cell debris through a process called phagocytosis and digest them in phagolysosomes, which results from the fusion of phagosomes with lysosomes. Lysosomes are membrane-enclosed organelles containing a set of hydrolases capable of breaking down the diverse classes of macromolecules. This degradation not only contributes to pathogen removal and clearance of an inflammatory site, for example, but also facilitates the presentation of resulting peptides to cells of the specific immune system, like T or B lymphocytes. This happens via presentation on major histocompatibility complex II (MHC II) molecules. Thereby, an antigen-specific immune response can be initiated. In addition, phagolysosome-associated enzymes facilitate the production of large amounts of reactive oxygen species (ROS) that function to effectively kill ingested pathogens.

Through the action of stimulatory factors, e.g., in the case of tissue injury, large numbers of inflammatory monocytes, macrophage precursors, are recruited from the bone marrow via chemokine gradients and various adhesion molecules. In such pathophysiological situations, the migrating (recruited) macrophages far outnumber the tissue-resident macrophages [9, 22]. Consequently, growth factors and cytokines released in the local tissue microenvironment force recruited and resident macrophages to proliferate and undergo substantial phenotypic and functional changes [34].

Depending on microenvironmental stimuli and the nature of the pathogen/antigen structure a macrophage encounters, differentiation into M1 or M2 macrophages could be favored. The predominant differentiation profile determines the functional orientation of the macrophages.

In this article emphasis is put on the (I) macrophage’s effector functions, (II) ROS produced, (III) the signaling pathways engaged, and (IV) the role of macrophage subpopulations in health and disease.

## Macrophage effector functions

As mentioned earlier, *phagocytosis* is an outstanding ability of macrophages and represents one of their main effector mechanisms. Macrophages effectively internalize particulate structures, with the uptake of pathogens being of particular relevance. The recognition and binding of such structures takes place through their opsonization by means of complement factors or antibodies. For this purpose, macrophages are equipped with specific receptors, some of which also represent macrophage-specific surface markers (immune cell surface markers are classified as *clusters of differentiation*, CDs). These are mainly the representatives of the complement receptors, CR1–CR4, as well as the so-called Fcγ receptors, FcγRI, FcγRII, and FcγRIII (Table 2). The Fcγ receptors bind with different affinity to the Fc tail of the immunoglobulin G (IgG) molecule, which is located opposite to the antigen-binding domains of IgG and, thus, still accessible when IgGs have specifically bound to, e.g., the surface of pathogens, thereby enabling their phagocytosis. This process is known as antibody-dependent cellular phagocytosis (ADCP). Ideally, this process leads to ROS-mediated killing and enzymatic digestion of pathogens or other particulate matter in the phagolysosome. Peptide fragments consisting of 12–25 amino acids that originate from protein (pathogen) digestion can be loaded onto major histocompatibility complex (MHC) class II molecules when endoplasmic reticulum (ER)-derived vesicles containing newly synthesized MHC molecules fuse with phagolysosomes. Peptide (antigen)-loaded MHC class II molecules are then transported to the cell surface (Fig. 2), where they serve to activate cells of the specific immune system, in particular T helper cells (T_H_ cells), in an antigen-specific way.

**Table 2:** Macrophage receptors involved in phagocytosis

Fcγ receptor ligand affinity	Ligand	Affinity
FcγRI (CD64)	IgG1, IgG3 (IgG4, IgG2)	High
FcγRII (CD32)	IgG1, IgG3 (IgG4, IgG2)	Low
FcγRIII (CD16)	IgG1, IgG3	Low

Complement receptor	Ligand/affinity
CR1 (CD35)	C3b > C4b > iC3b
CR2 (CD21)	C3d/C3g > iC3b
CR3 (CD11b/CD18)	iC3b; ICAM-1
CR4 (CD11c/CD18)	iC3b

**Fig. 3:**
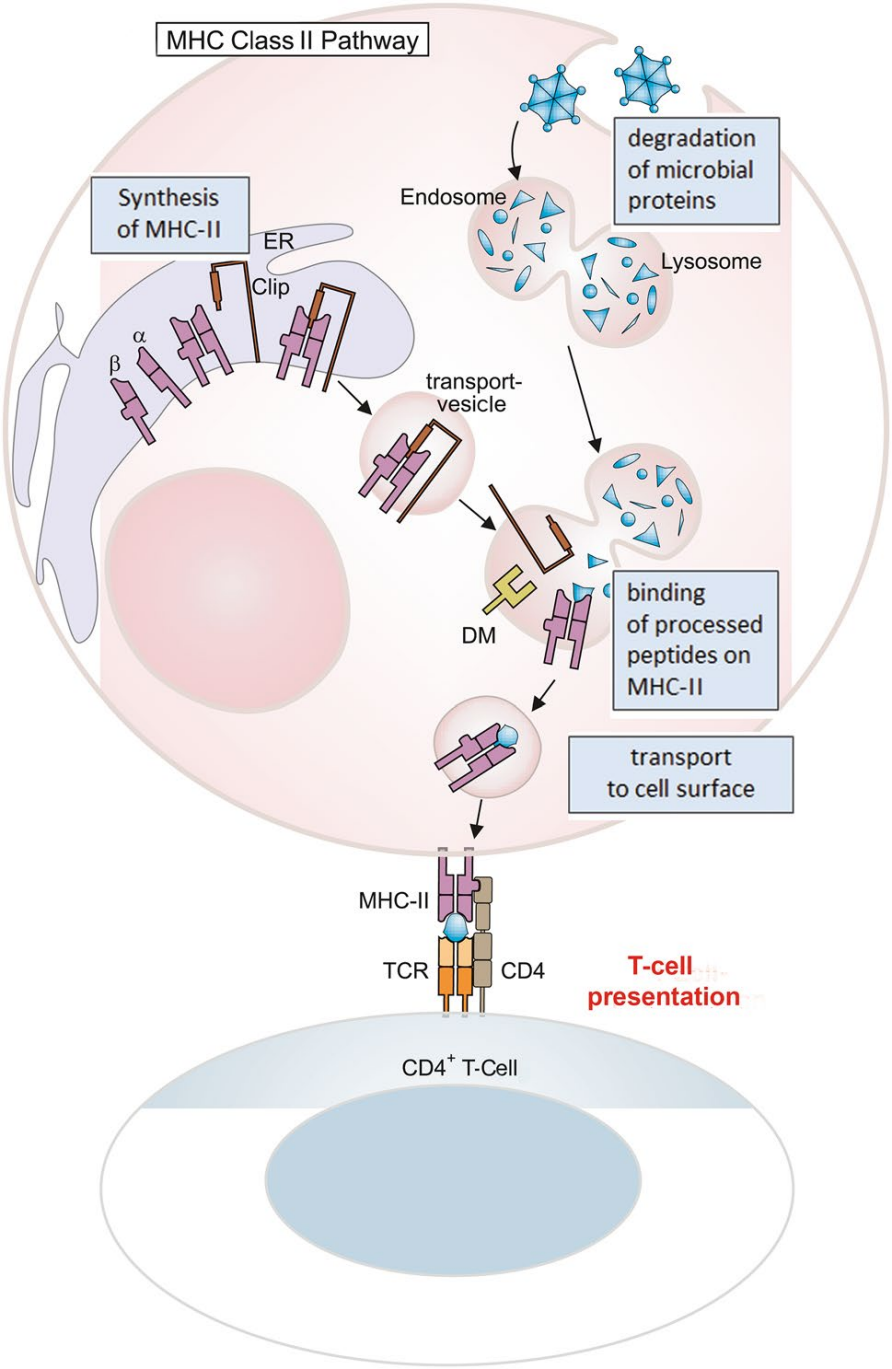
Processing of protein antigens: the MHC-II pathway. Protein antigens are taken up by antigen-presenting cells (APC) into endosomes, which then fuse with lysosomes. There the proteins are broken down into protein fragments. After their synthesis in the ER, MHC-II molecules are transported in vesicles. Newly synthesized MHC-II molecules carry a peptide that occupies the antigen binding site and is called CLIP (class II invariant chain peptide). If both vesicles fuse, CLIP is released from the peptide binding site of the MHC-II by the protein DM and MHC-II can now bind antigen fragments. These MHC-II/peptide complexes are then transported to the cell membrane and can be recognized by CD4-positive cells ([75] with kind permission)

*Immune cell recruitment and activation* represent two other major macrophage effector functions. One mechanism by which macrophages, whether recruited in response to a stimulus or tissue-resident ones, attract and activate other immune cells is the production and release of soluble factors which include proinflammatory cytokines such as interleukin-1β (IL-1β) and tumor necrosis factor alpha (TNFα), IL-6, IL-12, as well as chemokines like C-X-C motif chemokine ligand 8 (CXCL8, IL-8). This happens as soon as macrophages encounter pathogens/damage. Whereas IL-1β and TNFα cause, among other things, endothelial activation, increased vascular permeability, and activation of lymphocytes, IL-12 leads to the activation of natural killer (NK) cells, a type of cytotoxic lymphocyte critical to the innate immune system. NK cells function to eliminate virus-infected cells and cells infected by other intracellular pathogens, and tumor cells. Macrophages have typical receptor equipment for the effective recognition of pathogens. These so-called pattern recognition receptors (PRR), whose best-known representatives belong to the Toll-like receptors (TLR), recognize conserved structures that are characteristic of different classes of pathogens. Such typically pathogenic and therefore “foreign” structures are also referred to as pathogen-associated molecular patterns (PAMPS) and include, among others, double-stranded RNA, unmethylated CpG DNA, lipoproteins, and lipopolysaccharides (LPS) (Fig. 3).

**Fig. 4:**
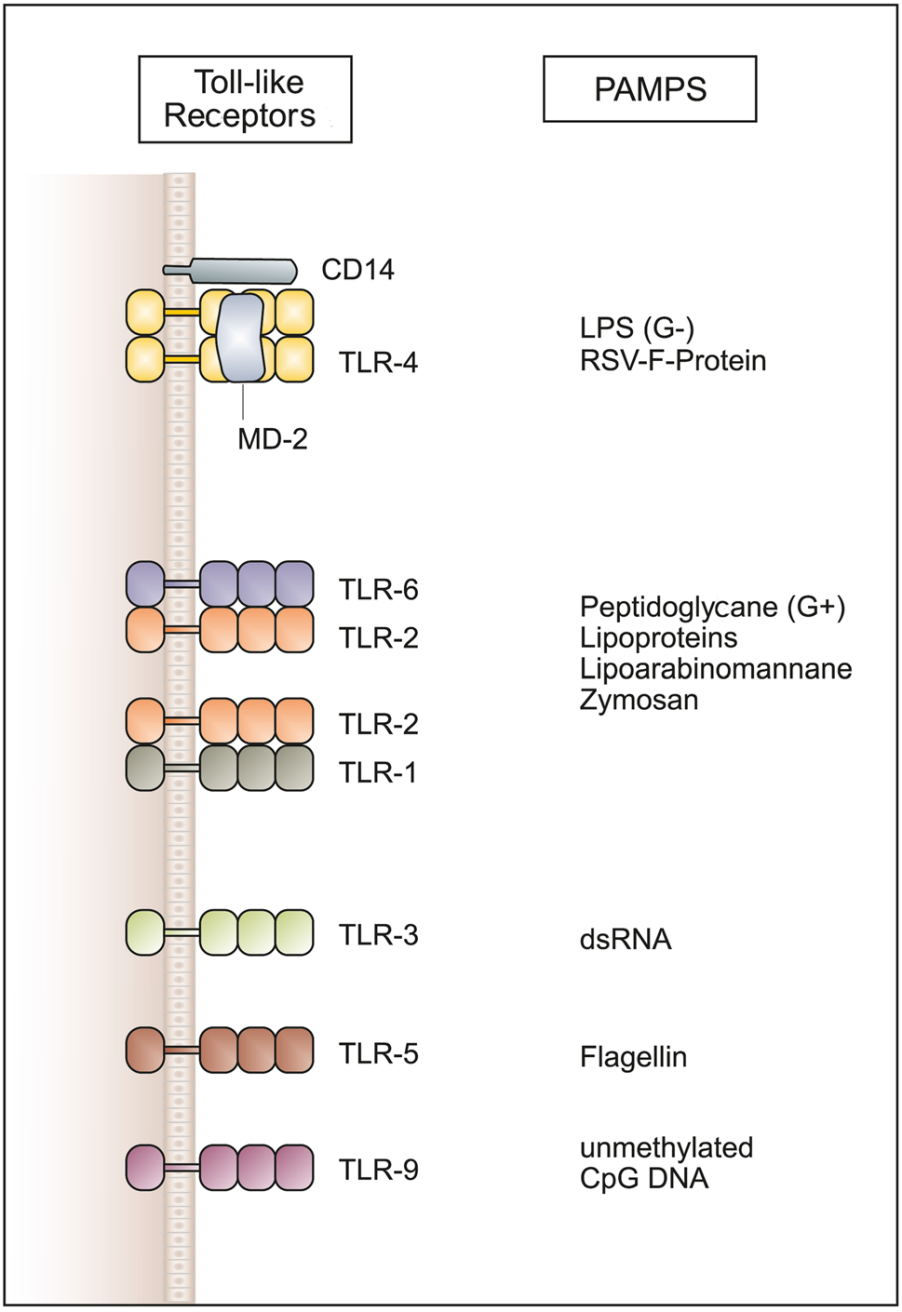
Toll-like receptors (TLRs) and their ligand specificity. Different TLRs and their ligand specificity. TLRs recognize a variety of pathogen-associated structures (PAMPs). The recognition of LPS by TLR-4 requires the accessory proteins CD14 and MD-2. TLR-2 recognizes very different ligands and interacts with TLR-6 and TLR-1. TLR-3 is involved in the recognition of viral double-stranded RNA (dsRNA), TLR-5 binds flagellin, a protein of bacterial flagella, and TLR-9 recognizes bacterial DNA on the basis of its unmethylated CpG motifs ([76] with kind permission)

Activation of the TLRs by binding PAMPs triggers the probably most typical signaling pathway of the nonspecific defense, the NF-κB signaling pathway, which ultimately leads to the production/release of the aforementioned cytokines (Fig. 4). Macrophage activation via TLRs, but also by, e.g., the macrophage-activating cytokine, interferon-γ, leads to increased phagocytotic activity and pathogen killing. The increased production of ROS/reactive nitrogen species (RNS) plays an important role in the effective killing of phagocytosed pathogens.

## Generation of ROS/RNS

The production of antimicrobial reactive oxygen and nitrogen species (ROS/RNS) is an important microbicidal mechanism of phagocytic cells. Congenital enzyme defects associated with a lack of or reduced activity of ROS/RNS-forming enzymes manifest themselves in the incomplete killing of pathogens or tumor cells and affected patients suffer from recurrent infections or cancer, respectively. ROS are generated intracellularly as by-products during various electron-transfer reactions, especially in the mitochondrial respiratory chain. However, receptor-mediated stimulation of ROS production is the key to important effector functions of macrophages. Various receptors, including complement receptors, TLRs, and receptors for bacterial peptides (e.g., the fMLF receptor) induce the production of ROS/RNS in phagolysosomes. Here, the assembly of the NADPH oxidase plays a dominant role. Its subunits p22 and gp91, which are located in the cell membrane and therefore in the phagolysosome, become assembled with the initially cytosolically located subunits (p40, p47, and p67) which provides the active NADPH oxidase. The formation of the active NADPH oxidase leads to an increase in oxygen consumption, which is referred to as “oxidative burst”. This creates superoxide anion radical (${\text{O}}_{2}^{ \cdot - }$) in the lumen of the phagolysosome, which is converted into H_2_O_2_ by superoxide dismutase (SOD) [59]. Further reactions lead to the formation of toxic ROS, including ${\text{OH}}^{ \cdot }$ and ${\text{OCl}}^{ - }$ from H_2_O_2_. The antimicrobial repertoire of macrophages also includes toxic nitrogen oxides, especially ${\text{NO}}^{ \cdot }$. In cells that produce both ${\text{O}}_{2}^{ \cdot - }$ and ${\text{NO}}^{ \cdot }$, such as macrophages, the near diffusion-limited reaction between ${\text{NO}}^{ \cdot }$ and ${\text{O}}_{2}^{ \cdot - }$ is likely to occur and yields peroxynitrite (${\text{ONOO}}^{ - }$) [17]. The peroxynitrite ion itself is not highly reactive; however, its acid form, peroxynitrous acid (ONOOH) is very unstable and reactive. Therefore, it is the formation of peroxynitrous acid that is considered as the mechanism underlying superoxide anion radical-mediated toxicity when Fenton chemistry is not at work [17]. ${\text{NO}}^{ \cdot }$ is produced in macrophages by the inducible NO synthase (iNOS) which in comparison to the forms constitutively expressed in other tissues, nNOS and eNOS, produces much larger amounts of ${\text{NO}}^{ \cdot }$. Increased expression/activity of iNOS and the resulting increased production of ${\text{NO}}^{ \cdot }$ have been associated with many pathophysiological processes, including trauma, inflammation, ischemia/hypoxia, and a severe drop in blood pressure as observed during septic shock. Mechanistically, ${\text{NO}}^{ \cdot }$ is formed by the oxidation of l-arginine which results in citrulline in addition to ${\text{NO}}^{ \cdot }$ as shown below:

$${\text{2 Arginine }} + {\text{ 3O}}_{{2}} + {\text{ 3 NADPH}} \to \;{\text{2 Citrulline }} + {\text{ 2NO}}^{ \cdot } + {\text{ 3 NADP}}^{ + } .$$

For this purpose, arginine can be withdrawn from the serum, where it is present at almost 100 µM concentrations, with the kidney being the most important source.

## Macrophage cell signaling

The acquisition/exercise of the various effector functions of macrophages requires the specific activation of signaling pathways in the cells. TLR ligands, cytokines, immunocomplexes, hypoxia and CD4-positive T lymphocytes, provisional extracellular matrix (ECM) and collagen remodeling and tissue structure are important stimuli that lead to the activation of macrophages [50], e.g., via Jak/STAT, NF-κB, and redox signaling.

### Cytokines and JAK/STAT signaling

Cytokines represent a large group of regulatory proteins that act primarily on the immune cell differentiation, activation, and function. This also applies to macrophages. Exposure to certain cytokines results in an adequate response of macrophages to various challenges such as infection, tissue damage, tumor, or alloantigen. This is achieved by a subtle balancing of production, release, or activation of a large set of individual cytokines whose effects are defined by complex synergistic or antagonistic interactions. Besides their actions on macrophages and other immune cells, cytokines potently affect other cells and tissues.

Members of the heterologous group of cytokines bind to different types of specific receptors, among them receptor kinases (e.g., TGF-β1 receptor) and tyrosine kinase-linked receptors that lack intrinsic kinase activity. The latter group of receptors is also referred to as cytokine receptors (e.g., IL-1β and TNFα receptor).

Ligand binding to these receptors by a wide variety of cytokines or growth factors generally provokes di- or trimerization of monomeric subunits, which in turn induces rapid activation of kinases associated with the receptor, typically members of the JAK (Janus kinase) family of tyrosine kinases. Phosphorylation of the receptor itself enables recruitment and binding via SH2 (src homology 2) domains of members of the STAT (signal transducers and activators of transcription) family of transcription factors to the receptor. This allows JAKs to phosphorylate STATs, which subsequently form dimers that are capable of entering the nucleus and specifically initiate transcription of target genes, which include cytokines, SOCS1–3, and transcription factors such as GATA3, C-Maf, c-myc, NFAT1/NFAT2 [30, 33].

### TLR/NF-κB signaling

**Fig. 5:**
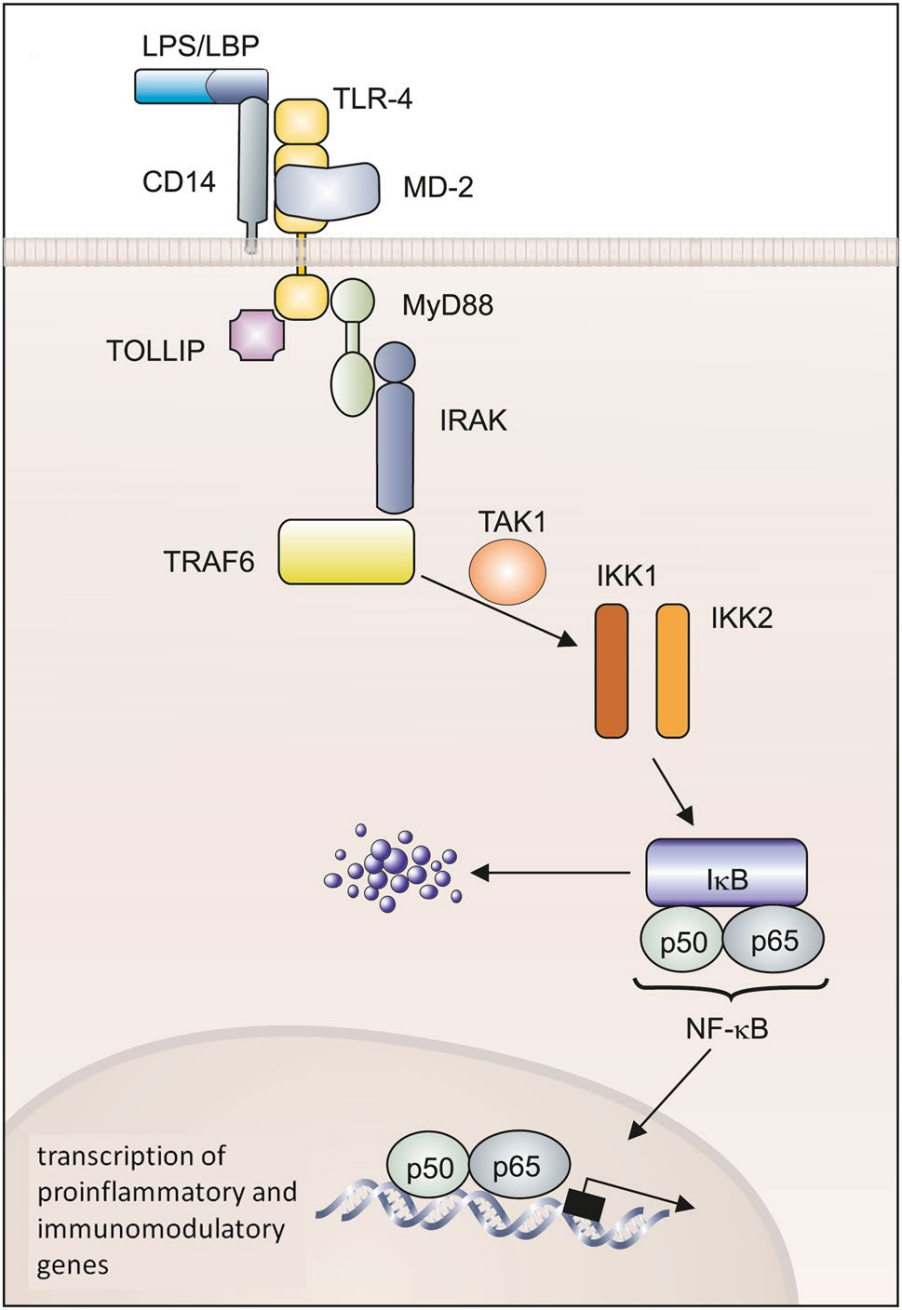
Signal transduction pathways of Toll-like receptors. The binding of the ligand (LPS/LBP/CD14; MD-2) to TLR leads to the association of adapter molecules such as MyD88, Toll-interacting Protein (TOLLIP), the protein kinase IRAK, and TRAF6 (TNF-receptor associated factor 6). TRAF6 activates IκB kinases 1 and 2 (IKK-1/2) via the kinase TAK1 (TGF-β activated kinase). These kinases phosphorylate IκB, which leads to the degradation of the inhibitory protein and releases NF-κB as a dimer. NF-κB migrates into the cell nucleus and induces a transcriptional activation of proinflammatory and immunomodulatory genes ([76] with kind permission)

The NF-κB family of transcription factors plays a crucial role in inflammatory and apoptotic responses. Family members include NF-κB1 p50, NF-κB2 p52, RELA (also called p65), RELB, and c-REL1 which as homodimers or heterodimers mediate transcription of NF-κB target genes [66]. They are retained in the cytoplasm by interaction with the inhibitory molecule IκB. Two NF-κB activation cascades can be discriminated. In the classical (canonical) activation pathway, in response to various signals IκB becomes phosphorylated by serine protein kinases, mainly IKK2 but also IKK1. IκB is then degraded in the proteasome, facilitating the translocation into the nucleus of p50/p65 NFκB. This heterodimer is the most abundant complex, often referred to as being “NF-κB”. It initiates and enhances the expression of target genes including iNOS, the chemoattractant cytokine, IL-8, the inflammatory TNFα, IL-1β, and IL-6, and adhesion molecules (ICAM1) and chemokines (IP-10, MCP-1, MIP-1; Fig. 5, for details see [45]). In the alternative activation cascade, IKK1 mediates processing of p100 to p52, the latter forming heterodimers with relB which upon transfer into the nucleus initiate transcription of NF-κB target genes. C-Rel is of special importance for, e.g., the transcriptional induction of IL-12 p40 in macrophages [38].

Some stimuli preferentially activate the alternative pathway; others, including lymphotoxin-β (LTβ) and LPS, activate both cascades [10, 11]. It is supposed that the two NF-κB cascades are activated in a sequential manner: the early response mediated by p65 containing dimers, the latter then continues a response with relB-containing complexes to sustain the NF-κB activation [54].

Oxidative stress, viral proteins, growth factors (angiotensin II), mitogens, the inflammatory cytokines IL-1β and TNFα via IL1 /IL-18 receptors or TNF receptors, respectively, NOD-like receptors, and TLR ligands activate IKKβ [31]. Activation of all these receptors also feeds into other signaling pathways that interfere, directly or indirectly, with the NF-κB signaling pathway [14]. Upon activation by LPS, TLR4 through Myd88 activates NF-κB, but in addition activates mitogen-activated protein kinase (MAPK), IRF5 pathways, and via endosomal TLR4 signaling, IRF3 and the production of the strong antiviral interferons become induced [14]. Autocrine effects of any cytokines released from activated macrophages further add to the complexity of resulting responses.

In humans, at least ten different TLRs exist which differ in their specificity towards distinct and characteristic pathogenic structures, like LPS, double-stranded RNA or unmethylated CpG DNA, so-called pathogen-associated molecular patterns. For this reason, TLRs are also called PRRs (pattern recognition receptors). The first TLR to be identified is TLR4, which is activated by LPS (gram-negative bacteria). LPS is bound to the macrophage surface via LBP and is bound by TLR4 in this complex (Fig. 4).

### Redox signaling

Accumulating evidence supports a signaling role for ROS that are generated by macrophages during oxidative burst.

In the last few years the view has hardened that ROS—at least when present in certain concentrations—can be viewed as a second messenger. In full agreement with this function, ROS show certain characteristics such as stimulus-dependent transient induction (e.g., via the activation of NADPH oxidase), specific function (interfering specifically with signaling components like, e.g., kinases), and the rapid termination of the signal by antioxidant enzymes/antioxidants (catalase, superoxide dismutase, glutathione peroxidases) [18]. The modulation of the intracellular concentration of calcium [Ca^2+^]_i_ is a general mechanism involved here because many intracellular signaling pathways actually depend on it.

ROS have been shown to induce the activation of NF-κB, although the underlying mechanisms still remain to be elucidated fully [32]. Reversible S-glutathiolation has been established as one mechanism underlying the regulation of, e.g., the transcription factor activator protein 1 (AP1) [19].

It is well established that ROS produced by macrophages (${\text{O}}_{2}^{ \cdot - }$ and the species subsequently formed therefrom) contributes to the oxidation of low-density lipoproteins (LDL). Oxidized LDL is rapidly taken up by macrophages leading to their transformation into “foam cells”, which are associated with an increased risk for atherosclerosis and complications that are related to this. Notably, oxidized LDL itself has been shown to interfere with many signaling pathways in macrophages [41].

## M1/M2 macrophage polarization

Monocytes/macrophages are characterized by a strong plasticity and heterogeneity. Dependent on the surrounding micromilieu and the immunological context, macrophages can acquire distinct functional characteristics through a process called polarization. The polarization leads to maturation and formation of “classically activated” M1 or “alternatively activated” M2 macrophages, respectively, which represent major functional macrophage categories. The alternatively activated macrophages have been further subcategorized (Table 3). The M1 macrophages have been associated with enhanced phagocytotic activity, antigen-presenting capacity, and increased synthesis and release of proinflammatory cytokines. The cytokine interferon-γ (IFNγ) was the first identified substance converting resting macrophages into M1. Substances known to cause/promote the formation of M1 macrophages now include bacterial LPS and autocrine-acting IFNβ.

**Table 3:** Macrophage subtypes originating from their polarization [17, 18]

Macrophage type	Macrophage Subtype	Induction by	Surface markers	Cellular markers	Production of cytokines
M1 “classically activated”	M1	LPS, IFNγ, TNFα, GM-CSF	CD68, CD80, CD86, IL-1R, TLR2, TLR4, iNOS, IFNγR, MHC II^high^, Fc-RI/II/III	NF-κB, STAT1, STAT5, IRF3, IRF5	IL-1α, IL-1β, IL-6, IL-12, IL-18, TNFα, M-CSF
	Diabetes/obesity-associated (M1?)	Insulin resistance, persistent & hyper-inflammatory activation	BCA1, CD11c, CD36	PLIN2, Msr1A	
M2 “alternatively activated”	M2a	IL-4, IL-13	CD200, IL-1RII, Dectin-1, MHC II^low^	IRF4, PPARγ, STAT6	IL-10, IL-1Ra, TGFβ
	M2b	Immune complexes + TLR/IL-1β	CD86, MHC II^low^	IRF4, SOCS3	IL-1β, IL-6, IL-10, TNFα
	M2c	IL-10, TGFβ	CD163, CD206, TLR1	IRF4, SOCS3	IL-10, TGFβ

M1 macrophages exert strong cytotoxic activity against infected cells, remove pathogens during infection, and mediate resistance against infections. This is achieved by enhanced production and secretion of proinflammatory/activating cytokines, adhesion molecules, chemokines, and cyclooxygenase-2 (Cox2) as well as by increased production of ${\text{O}}_{2}^{ \cdot - }$ due to activation of NADPH oxidase [45, 64]. M1 macrophages may cause inflammation and tissue damage by these mechanisms, particularly if resolution of inflammation and initiation of tissue repair is not initiated or not initiated in time.

The polarization towards M2 cells, on the other hand, is mediated by the cytokines IL-4 and IL-13. M2 macrophages may promote angiogenesis and neovascularization, stromal activation and remodeling, tissue repair, fibrosis, and is fairly anti-inflammatory in general. In support of this view, IL-4 has been shown to inhibit oxidative burst and inflammatory cytokine expression, e.g., of IL-1β.

In the context of cancer, M2 macrophages have been associated with tumor progression and immunosuppression, thereby negatively affecting the prognosis of patients.

Different functional states of macrophages have been associated with cell shape changes [50]. Vice versa, by applying a micropatterning approach, macrophage cell shape could be directly controlled, demonstrating that elongation per se, without any exogeneous cytokines is able to induce the expression of M2 makers (Table 3) such as arginase-1 or CD206 [50].

It is becoming increasingly clear that the M1/M2 polarization also affects the macrophage metabolism. Metabolic disorders such as type 2 diabetes mellitus (T2DM) are therefore associated with drastic consequences for macrophage polarization and function. Classically activated, proinflammatory M1 macrophages depend to a large extent on glycolysis and produce lactate as the tricarboxylic acid cycle is blocked at two steps. Alternatively activated M2 macrophages prefer β-oxidation and oxidative phosphorylation to synthesize ATP (for an excellent review, see [61]). It is well established that continuous and excessive inflammatory stimuli, e.g., in various inflammatory and metabolic diseases, including T2DM, lead to macrophage activation. In the situation of T2DM, a particulate activation state arises, which is defined by expression of specific markers that include macrophage scavenger receptor (MSR1), ATP-binding cassette subfamily A member 1 (ABCA1), perilipin-2 (PLIN2), in addition with CD11b and CD36 [5, 39, 61].

Macrophages are also considered as key modulators in cancer progression. Infiltration of macrophages in and around tumors (tumor-associated macrophages, TAMs) is regularly observed and their presence is associated with poor prognosis in solid tumors [13, 23]. The interaction of TAMs with either tumor cells or the tumor microenvironment drives macrophage polarization. For a long time it was assumed that TAMs originate from circulating monocytes [48]. In a murine mammary carcinoma model, tumor growth was accompanied by loss of resident cells and repopulation of the TAMs by monocytes [20]. TAMs generally exhibit an M2 phenotype [2]. In contrast, macrophages of embryonic origin persisted in pancreatic ductal adenocarcinoma and significantly contributed to disease progression and fibrosis [74]. Although they have no cytotoxic activity themselves, they produce growth factors for tumor cells and exhibit immunosuppressive activity [2]. M2 macrophages may promote angiogenesis and neovascularization, remodeling of stroma and the tumor niche, and dissemination of tumor cells all contributing to tumor progression. Fast-growing solid tumors typically have only insufficient oxygen supply resulting in hypoxic zones, especially in the tumor center or in perinecrotic areas. Mechanistically, this hypoxia via induction of, e.g., IL4 and IL-10, contributes to the recruitment and M2 polarization of macrophages. TAMs, therefore, preferentially localize in these hypoxic areas of a tumor [35]. The M1 phenotype functions in phagocytosis and lysis of tumor cells [43]. Through their enhanced antigen-presentation ability, M1 macrophages enable cytotoxic leukocyte functions. Increased production and release of inflammatory cytokines such as TNFα, IL-6, or IL-12 contribute to these effects and may support tumor cell apoptosis [43].

There is ample evidence that the location of macrophages in and around the tumor is critical to the polarization and, thus, function of macrophages. Location-related signals enforce distinct macrophage functions. In this sense, M2 macrophages promote tumor cell motility and invasion, favor metastasis, and stimulate neoangiogenesis [28, 42, 43, 57]. Most notably, the tumor-related distribution pattern of macrophages has been identified as an independent prognostic factor in gastric cancer [44].

## Macrophages as therapeutic targets

The involvement of macrophages/TAMs in the aforementioned processes such as cellular homeostasis, repair mechanisms, regeneration, and angiogenesis make macrophages a promising target, e.g., in tumor therapy. Of high relevance for patients with cancer is the fact that TAMs impair the effectiveness of chemotherapeutic agents, radiation, and angiogenesis inhibitors [13, 15, 60]. As stated earlier, the tumor environment, including tissue fibrosis, hypoxia, availability of nutrients, and lymphocyte-derived factors all determine the macrophage phenotype [13]. TAMs are thought to generally display an M2-like phenotype [2]. However, recent data suggest that TAMs show an elevated degree of heterogeneity. This could be observed both between different patients with tumors and between different tumor lesions in a patient. Accordingly, TAMs can acquire a whole spectrum of phenotypic and functional profiles in response to environmental stimuli [70]. Accordingly, specific TAM subpopulations support oncogenesis via diverse mechanism which include stimulation of angiogenesis, matrix degradation, and resistance to therapy; others seems to support the efficacy of various anticancer (immuno)therapies [1, 48, 70]. TAMs that are located in perivascular or hypoxic areas exhibit a rather M2-like phenotype and show proangiogenic, profibrotic, and immunosuppressive properties [40]. In contrast, TAMs located at the tumor invasion site often show a proinflammatory M1-like phenotype and display rather tumoricidal properties [72].

It was assumed that TAMs are predominantly derived from circulating monocytes [53, 65], but up to 50% of TAMs in murine models of lung, brain, and pancreatic cancer have been shown to originate from tissue-resident macrophages [3, 47, 74]. A promising therapy is to stop the replenishment of TAMs from circulating monocytes. As the latter strongly depends on CCL2-CCR2 signal transduction for their mobilization from the bone marrow and the recruitment to the tumor tissue, CCR2 blockade actually improved the efficacy of chemotherapy, radiation therapy, and immunotherapy [8, 21, 37, 55, 62]. In a similar approach, these effects are associated with increased T cell infiltration, a finding that was also observed in some preclinical models. Therefore, further combinations with checkpoint immunotherapy are currently under investigation.

With another strategy, namely the inhibition of the CSF1/CSF1R axis, the apoptotic death of TAMs is counteracted. Indeed, CSF1R inhibition improved T cell responses in combination with chemotherapy or radiation therapy in several animal models [12, 51, 71].

## The dark side of macrophages

Because of the great heterogeneity and plasticity that macrophages exhibit it is not surprising that they are involved in a multitude of functions and processes ranging from the defense against pathogens, the clearance of old, senescent, or dead cells, maintenance of tissue homeostasis and mediating fibrosis, tissue repair, and regeneration, tumor growth, and immunosuppression. In the traditional view the M2 macrophage phenotype is pro-regenerative and associated with positive wound healing outcomes, whereas the M1 phenotype is proinflammatory and associated with pathogenesis. According to today’s understanding, this is more complex: both M1 and M2 macrophages play different, but equally vital, roles in organizing/mediating tissue repair [49]. As explained earlier, for the therapy of cancer, the immunosuppressive, tumor growth-promoting M2 phenotype should be overcome and the inflammatory tumoricidal M1 phenotype should be supported. Any missing, delayed, or incomplete termination of an inflammatory reaction against pathogens, cell debris, or the like can lead to fatal adverse effects from macrophages. Failure to appropriately terminate an inflammatory response, in the worst case, can lead to an excessive recruitment/activation of immune cells and the excessive production and release of cytokines, also referred to as “cytokine storm” or “cytokine release syndrome” (CRS) [67]. As currently defined, cytokine storm is a condition associated with elevated systemic cytokine levels, systemic clinical signs of inflammation, and severe secondary tissue damage [16]. As excellently reviewed in [16], a cytokine storm can be induced by pathogenic invasive infectious disease [58, 73] or as a side effect of chimeric antigen receptor T-cell (CAR-T) cell therapy [27, 52], a recent cancer immunotherapy approach. Depending on the triggering agent, the induced cytokine profile can be of different composition [67]. While, e.g., CRS caused by SARS leads to the predominant release of IL-1β, IL-6, IL-12, IFNγ, IP10, and MCP-1 [6], COVID-19-associated CRS [56] has been characterized by increased release of IL-2, IL-7, IL-10, G-CSF, IP10, MCP-1, MIP-1α, and TNFα [29]. If pathogens are involved, typically PRRs and NF-κB signaling play an essential role. Therefore, macrophages and macrophage-derived cytokines such as MCP-1, MIP-1α, TNFα, and IL-8 regularly contribute to the development of a cytokine storm.

## Glossary

B lymphocytes: A type of white blood cell of the lymphocyte subtype, upon activation by an antigen, a naïve or memory B cell proliferates and differentiates into an antibody-secreting effector cell, known as a plasma cell.
CD molecules: Cell surface molecules, which are expressed on various types of cells in the immune system that are designated by the cluster of differentiation or CD number.
C-Maf: (Proto-Oncogene C-Maf), a DNA-binding, leucine zipper-containing transcription factor that acts as a transcriptional activator or repressor; among its related pathways is NFAT-dependent transcription in lymphocytes.
c-myc: (MYC Proto-Oncogene), a member of basic helix-loop-helix (BHLH) transcription factors.
Complement: (Also referred to as complement cascade), a system of serum and cell surface-bound proteins that is a part of the immune system and functions to enhance (complement) the ability of antibodies and phagocytic cells to remove microbes and damaged cells from an organism, promote inflammation, and attack a pathogen’s cell membrane.
c-Rel: (Proto-Oncogene C-Rel), a member of the NF-κB family of transcription factors that contains a Rel homology domain (RHD) and two C-terminal transactivation domains.
CRS: Cytokine release syndrome; can be triggered by a variety of factors such as infections and occurs when large numbers of leukocytes are activated and release inflammatory cytokines, which in turn recruit and activate even more leukocytes.
Fc receptor: A cell surface receptor specific for the C-terminal region of an immunoglobulin (Ig); there are several types of Fc receptors including those specific for different IgG isotypes, FcγRI-III, that are implicated in, among other processes, phagocytosis.
GATA3: Member 3 of a family of transcription factors characterized by their ability to bind to the DNA sequence GATA.
ICAM1: Intercellular adhesion molecule 1.
IP-10: Alpha chemokine.
Major histocompatibility complex II (MHC II): A class of major histocompatibility complex (MHC) molecules normally found only on professional antigen-presenting cells such as macrophages; antigens presented by class II peptides (e.g., to T lymphocytes) are derived from extracellular proteins.
MCP-1: Monocyte chemotactic and activating factor.
CCL2: C-C motif chemokine ligand 2.
CCL3: C-C motif chemokine ligand 3.
NFAT: Nuclear factor of activated T cells; a family of transcription factors shown to be important in immune response.
NOD-like receptors: Nucleotide-binding oligomerization domain-like receptors; type of pattern recognition receptor.
Opsonization: The important process in host defense by which pathogens, particles, or complexes are made readily ingestible for uptake by phagocytic cells.
Pluripotent hematopoietic stem cells: Undifferentiated cells that can self-renew and potentially differentiate into all hematopoietic lineages including hematopoietic stem cells and progenitor cells.
SARS: Severe acute respiratory syndrome; viral respiratory disease caused by severe acute respiratory syndrome coronavirus (SARS-CoV or SARS-CoV-1), which caused the 2002–2004 SARS outbreak.
SOCS: (Cytokine-inducible SH2-containing protein), a family of proteins involved in inhibiting the JAK-STAT signaling pathway.
T lymphocytes: A type of white blood cell of the lymphocyte subtype; CD8^+^ T cells, also known as cytotoxic T cells, are able to directly kill virus-infected cells, as well as cancer cells. CD4^+^ T cells function as “helper cells”: they recognize antigen peptides presented by MHC II and function by further activating memory B cells and cytotoxic T cells, which leads to a larger immune response.
